# Urinary arsenic, pesticides, heavy metals, phthalates, polyaromatic hydrocarbons, and polyfluoroalkyl compounds are associated with sleep troubles in adults: USA NHANES, 2005–2006

**DOI:** 10.1007/s11356-016-8054-6

**Published:** 2016-11-17

**Authors:** Ivy Shiue

**Affiliations:** 1 Faculty of Health and Life Sciences, Northumbria University, Benton, Newcastle upon Tyne, England NE7 7XA UK; 2 Owens Institute for Behavioral Research, University of Georgia, Athens, GA USA

**Keywords:** Sleep, Arsenic, Pesticide, Heavy metal, Phthalate, Phenol, Polyfluoroalkyl, Hydrocarbon, Environmental chemical

## Abstract

Links between environmental chemicals and human health have emerged, but the effects on sleep health were less studied. Therefore, the aim of the present study was to investigate the relationships of different sets of environmental chemicals and common sleep troubles in a national and population-based setting. Data were retrieved from the United States National Health and Nutrition Examination Surveys, 2005–2006 including demographics, serum measurements, lifestyle factors, self-reported sleep troubles, and urinary environmental chemical concentrations. Statistical analyses including descriptive statistics, *t*-test, chi-square test, and survey-weighted logistic regression models were performed. Of all 5563 Americans aged 18–85, 2331 (42.0%) had wake-up at night, 2914 (52.5%) felt unrested during the day, 740 (13.4%) had leg jerks while sleeping, and 1059 (19.1%) had leg cramps for 2+ times a month. Higher levels of urinary arsenic, phthalates, and polyfluoroalkyl compounds were associated with wake-up at night. Higher levels of urinary 4-*tert*-octylphenol and polyfluoroalkyl compounds were associated with being unrested during the day. Higher levels of urinary arsenic, polyaromatic hydrocarbons, and polyfluoroalkyl compounds were associated with leg jerks while sleeping. Higher levels of urinary pesticides, heavy metals, phthalates, and polyaromatic hydrocarbons were associated with leg cramps while sleeping. However, there were no significant associations with other environmental chemicals such as parabens, bisphenol A, benzophenone-3, triclosan, perchlorate, nitrate, or thiocyanate. Eliminating arsenic, heavy metals, phthalate, pesticides, polyaromatic hydrocarbons, and polyfluoroalkyl compounds to improve sleep health might be considered while understanding the biological pathway with a longitudinal or experimental approach in future research would be suggested.

## Introduction

### Evidence before this study

Links between environmental chemicals and human health in the American adults including hypertension, cardiovascular disease, food allergy, oral health, emotional support, and cognitive function have emerged (Shiue 2015a, b, c, d, 2014a, b, 2013a, b, c), but the effects on sleep health, such as time to fall asleep as one of the indicators, were relatively less studied. Epidemiological studies have shown that the prevalence of sleep disturbances lies between 20 and 30% and increases with age, particularly in female (Zeitlhofer et al. 2000). Among the American adults, in studying risk contributors for sleep health it was observed that vitamin D level, a hormone that interacts with intranuclear receptors to effect transcriptional changes in many cell types including those in gut, bone, breast, prostate, brain, skeletal muscle, and the immune system (McCarty et al. 2014), could also play an important role in time to fall asleep at bedtime (Shiue 2013d).

### Study aim

However, it is unclear whether environmental chemicals in addition to sun radiation could influence sleep patterns as well. Following this context, therefore, the aim of the present study was to investigate the relationships of different sets of environmental chemicals and some common sleep troubles in a national and population-based setting using an independent dataset.

## Methods

### Study sample

As described online (more details via http://www.cdc.gov/nchs/nhanes.htm), the United States National Health and Nutrition Examination Surveys (NHANES) has been a national, population-based, multi-year, cross-sectional study since the 1980s. Study samples are representative of the civilian, non-institutionalized US population. Information on demographics, serum measurements, lifestyle factors, sleep pattern (Question: How does it usually take to fall asleep at bedtime? More details via http://wwwn.cdc.gov/Nchs/Nhanes/2005-2006/SLQ_D.htm) and urinary environmental chemical concentrations was obtained by household interview. In the current analysis, the 2005–2006 cohort as the most recent study cohort with all the required information mentioned above was selected for statistical analysis (more details via http://wwwn.cdc.gov/nchs/nhanes/search/nhanes05_06.aspx). Informed consents were obtained from participating subjects by the NHANES researchers.

### Biomonitoring

Urines were only collected in a representative subsample within 10 days of the household interview, being one third of the whole study cohort (more details via http://www.cdc.gov/nchs/data/nhanes/nhanes_05_06/lab_d.pdf), to measure environmental chemical concentrations in urine among people aged 6 and above (more details via http://www.cdc.gov/nchs/data/nhanes/nhanes_03_04/environmentalhealth_03.pdf). Urine specimens were processed, stored under appropriate frozen (−20 °C) conditions, and shipped to the Division of Environmental Health Laboratory Sciences, National Center for Environmental Health, Centers for Disease Control and Prevention for analysis.

The inductively coupled plasma-mass spectrometry (ICP-MS) method (Mulligan et al. 1990) was used to measure the following 12 elements in urine: beryllium (Be), cobalt (Co), molybdenum (Mo), cadmium (Cd), antimony (Sb), cesium (Cs), tungsten (W), tin (Sn), strontium (Sr), manganese (Mn) thallium (TI), lead (Pb), and uranium (U). Urine samples are diluted 1 + 9 with 2% (*v*/*v*), double-distilled, concentrated nitric acid containing both iridium (Ir) and rhodium (Rh) for multi-internal standardization (more details via http://wwwn.cdc.gov/Nchs/Nhanes/2005-2006/UHM_D.htm). The test principle utilized high-performance liquid chromatography-electrospray ionization-tandem mass spectrometry (HPLC-ESI-MS/MS) for the quantitative detection in urine of the following phthalate metabolites: monomethyl phthalate (MMP), monoethyl phthalate (MEP), monobutyl phthalate (MBP), monoisobutyl phthalate (MIBP), mono(3-carboxypropyl) phthalate (MCPP), mono(2-ethylhexyl) phthalate (MEHP), monobenzyl phthalate (MBzP), monoisononyl phthalate (MNP), mono(2-ethyl-5-oxohexyl) phthalate (MEOHP), mono(2-ethyl-5-hydroxyhexyl) phthalate (MEHHP), mono(2-ethyl-5-carboxypentyl) phthalate (MECPP), monocarboxyoctyl phthalate (MCOP), monocarboxynonyl phthalate (MCNP), and cyclohexane-1,2-dicarboxylic acid-mono(hydroxy-isononyl) ester (MHNCH). Urine samples are processed using enzymatic deconjugation of the glucuronidated metabolites followed by on-line solid phase extraction (SPE) coupled with reversed phase HPLC-ESI-MS/MS. Assay precision is improved by incorporating isotopically labeled internal standards of the phthalate metabolites and MHNCH (more details via http://wwwn.cdc.gov/Nchs/Nhanes/2005-2006/PHTHTE_D.htm).

Total and specific urine arsenic concentrations were determined by using inductively coupled-plasma dynamic reaction cell-mass spectrometry (ICP-DRC-MS) (more details via http://wwwn.cdc.gov/Nchs/Nhanes/2005-2006/UAS_D.htm) Urine is analyzed because urinary excretion is the major pathway for eliminating arsenic from the mammalian body. The method used on-line SPE coupled to HPLC and tandem mass spectrometry (HPL/CMS/MS). A sensitive method, SPE coupled on-line to HPLC and tandem mass spectrometry (MS/MS), was used for measuring bisphenol A (BPA), 4-*tert*-octylphenol (tOP), benzophenone-3 (BP-3), one chlorophenols triclosan, four parabens, and pesticides (more details via http://wwwn.cdc.gov/Nchs/Nhanes/2005-2006/EPH_D.htm and http://wwwn.cdc.gov/Nchs/Nhanes/2005-2006/PP_D.htm). A quantitative procedure was used for the measurement of nitrate, perchlorate, and thiocyanate in human urine using ion chromatography coupled with electrospray tandem mass spectrometry. Chromatographic separation is achieved using an IonPac AS16 column with sodium hydroxide as the eluant. The eluant from the column is ionized using an electrospray interface to generate and transmit negative ions into the mass spectrometer (more details via http://wwwn.cdc.gov/Nchs/Nhanes/2005-2006/PTH_D.htm).

To detect and measure metabolites of polyaromatic hydrocarbons (more details via http://wwwn.cdc.gov/Nchs/Nhanes/2005-2006/PAH_D.htm), the procedure involved enzymatic hydrolysis of glucuronidated/sulfated OH-polyaromatic hydrocarbons metabolites in urine, extraction, derivatization, and analysis using isotope dilution capillary gas chromatography tandem mass spectrometry (GC-MS/MS). Ion transitions specific to each analyte and carbon-13 labeled internal standards are monitored, and the abundances of each ion are measured. Moreover, solid phase extraction coupled to high-performance liquid chromatography-turbo ion spray ionization-tandem mass spectrometry (online SPE-HPLC-TIS-MS/MS) was used for the quantitative detection of perfluorooctane sulfonamide (PFOSA), 2-(*N*-methyl-perfluorooctane sulfonamido) acetic acid (Me-PFOSA-AcOH), 2-(*N*-ethyl-perfluorooctane sulfonamido) acetic acid (Et-PFOSA-AcOH), perfluorobutane sulfonate (PFBuS), perfluorohexane sulfonate (PFHxS), perfluorooctane sulfonate (PFOS), perfluoroheptanoate (PFHpA), perfluorooctanoate (PFOA), perfluorononanoate (PFNA), perfluorodecanoate (PFDeA), perfluoroundecanoate (PFUA), and perfluorododecanoate (PFDoA) (more details via http://wwwn.cdc.gov/Nchs/Nhanes/2005-2006/PFC_D.htm).

### Statistical analysis

Americans aged 18–85 with all the required study variables were included in the current statistical analysis. Urinary environmental chemical concentrations were all log transformed because they were highly skewed to one side. Associations of urinary environmental chemical concentrations (*x* variables) and the five common sleep troubles in the past month (*y* variables; more details via http://wwwn.cdc.gov/Nchs/Nhanes/2005-2006/SLQ_D.htm) were examined by using survey-weighted logistic regression models, with *P* < 0.05 considered statistically significant. Covariates including urinary creatinine, age, sex, ratio of family income to poverty (proxy of socioeconomic status), body mass index, serum cotinine (biomarker of smoking status), vitamin D levels (more details via http://wwwn.cdc.gov/Nchs/Nhanes/2005-2006/VID_D.htm), and physical activity level were adjusted. Statistical software STATA version 13.0 (STATA, College Station, Texas, USA; more details via http://www.stata.com/) was used to conduct all the analyses.

### Ethics concerns

Since there are only secondary data analyses employed without any participant personal information identified by extracting statistical data from the NHANES website in the present study, no further ethics approval for conducting the present study is required (more details via http://www.ethicsguidebook.ac.uk/Secondary-analysis-106).

## Results

Table 1 shows characteristics of the included study participants. Of all 5563 Americans aged 18–85, 2331 (42.0%) had wake-up at night, 2914 (52.5%) felt unrested during the day, 740 (13.4%) had leg jerks while sleeping, and 1059 (19.1%) had leg cramps while sleeping for 2+ times a month. Table 2 lists the frequency of common sleep troubles in the study participants. Tables 3, 4, 5, and 6 present the associations between urinary chemical concentrations and common sleep troubles, except for feeling overly sleeping during the day with no association found, accordingly. To be specific, higher levels of urinary arsenic, phthalates, and polyfluoroalkyl compounds were associated with wake-up at night. Higher levels of urinary 4-*tert*-octylphenol and polyfluoroalkyl compounds were associated with being unrested during the day. Higher levels of urinary arsenic, polyaromatic hydrocarbons, and polyfluoroalkyl compounds were associated with leg jerks while sleeping. Higher levels of urinary pesticides, heavy metals, phthalates, and polyaromatic hydrocarbons were associated with leg cramps while sleeping. There were no significant associations with other environmental chemicals such as parabens, bisphenol A, benzophenone-3, triclosan, perchlorate, nitrate, or thiocyanate (data not shown).

**Table 1:** Characteristics of the study participants aged 18–85 (*N* = 5563)

	*N* (%) or mean ± SD
Age	45.2 ± 20.3
18–39	2507 (45.1)
40–59	1486 (26.7)
60–85	1570 (28.2)
Sex	
Male	2675 (48.1)
Female	2888 (51.9)
Body mass index	28.5 ± 6.8
<18.5	101 (1.9)
18.5–24.9	1620 (30.9)
25–29.9	1734 (33.1)
30+	1782 (34.0)
Ratio of family income to poverty	
0–4.9	4318 (82.0%)
5+	951 (18.1%)
Serum cotinine (ng/mL)	55.8 ± 121.3
Serum vitamin D (ng/mL)	21.3 ± 9.4
Alcohol status	
≥12 drinks	2994 (68.8%)
Less than 12 drinks	1355 (31.2%)
Physical activity level	
Engaging moderately	3026 (54.4%)
Not moderately	2535 (45.6%)

**Table 2:** Characteristics of sleep troubles in the study participants aged 18–85 (*N* = 5563)

	Number (%)
Wake-up at night	
Never	2134 (38.4)
1 time a month	1091 (19.6)
2–4 times a month	1269 (22.8)
5–15 times a month	670 (12.0)
16–30 times a month	392 (7.1)
Unrested during the day	
Never	1746 (31.4)
1 time a month	893 (16.1)
2–4 times a month	1546 (27.8)
5–15 times a month	848 (15.2)
16–30 times a month	520 (9.4)
Overly sleepy during the day	
Never	1908 (34.3)
1 time a month	1180 (21.2)
2–4 times a month	1450 (26.1)
5–15 times a month	663 (11.9)
16–30 times a month	352 (6.3)
Leg jerks while sleeping	
Never	4389 (78.9)
1 time a month	391 (7.0)
2–4 times a month	451 (8.1)
5–15 times a month	175 (3.2)
16–30 times a month	114 (2.1)
Leg cramps while sleeping	
Never	3833 (68.9)
1 time a month	663 (11.9)
2–4 times a month	720 (12.9)
5–15 times a month	236 (4.2)
16–30 times a month	103 (1.9)

**Table 3:** Associations between urinary chemicals and wake-up at night in adults

	No wake-up at night (*n* = 3225, 58.1%)	Wake-up at night (*n* = 2331, 42.0%)	OR (95% CI)	*P* value
Arsenic (*n* = 1581, μg/L)				
Total arsenic	23.5 ± 73.7	22.2 ± 51.9	1.08 (0.97–1.19)	0.136
Arsenous acid	0.9 ± 0.7	0.9 ± 0.3	0.79 (0.31–2.01)	0.607
Arsenic acid	0.7 ± 0.3	0.8 ± 0.5	*1.86 (0.99–3.48)*	*0.053*
Arsenobetaine	12.7 ± 59.2	12.4 ± 41.8	1.03 (0.94–1.14)	0.476
Arsenocholine	0.4 ± 0.3	0.5 ± 0.3	1.05 (0.68–1.62)	0.805
Dimethylarsonic acid	5.8 ± 9.6	5.7 ± 7.9	1.03 (0.87–1.22)	0.715
Monomethylarsonic acid	1.0 ± 1.1	1.0 ± 1.2	1.08 (0.74–1.56)	0.681
Trimethylarsine oxide	0.7 ± 0.2	0.7 ± 0.2	1.51 (1.29–1.76)	<0.001
Phthalate (*n* = 1488, μg/mL)				
Mono(carboxyoctyl)	16.4 ± 111.7	16.9 ± 73.2	1.00 (0.90–1.13)	0.936
Mono(carboxynonyl)	5.5 ± 16.2	6.7 ± 31.1	0.96 (0.83–1.10)	0.514
Mono-2-ethyl-5-carboxypentyl	112.0 ± 356.7	93.9 ± 235.2	0.90 (0.80–1.00)	0.048
Mono-*n*-butyl	48.4 ± 338.5	35.0 ± 45.3	0.97 (0.85–1.10)	0.625
Mono(3-carboxypropyl)	4.3 ± 15.7	4.1 ± 10.0	1.01 (0.91–1.13)	0.813
Monoethyl	433.8 ± 1084.7	508.1 ± 1253.9	1.04 (0.94–1.14)	0.432
Mono(2-ethyl-5-hydroxyhexyl)	84.7 ± 308.3	75.0 ± 211.9	0.91 (0.82–1.01)	0.086
Mono(2-ethyl)-hexyl	13.2 ± 71.9	10.0 ± 31.4	0.94 (0.83–1.07)	0.330
Monoisobutyl	26.2 ± 477.4	11.1 ± 19.9	0.96 (0.84–1.10)	0.541
Mono-*n*-methyl	3.5 ± 12.9	5.5 ± 37.7	1.05 (0.95–1.16)	0.290
Monoisononyl	2.0 ± 8.6	1.8 ± 5.5	1.15 (0.92–1.44)	0.193
Mono(2-ethyl-5-oxohexyl)	53.0 ± 199.4	45.7 ± 124.5	0.91 (0.81–1.02)	0.096
Monobenzyl	35.4 ± 542.2	17.6 ± 33.3	0.96 (0.85–1.09)	0.508
Monocyclohexyl	0.5 ± 0.5	0.5 ± 1.1	1.88 (1.02–3.46)	0.044
Polyfluoroalkyl compounds (*n* = 1591, μg/mL)				
Perfluorooctanoic acid	4.5 ± 3.3	4.5 ± 3.7	0.95 (0.74–1.21)	0.649
Perfluorooctane sulfonic acid	21.0 ± 15.9	22.3 ± 20.9	0.99 (0.83–1.20)	0.947
Perfluorohexane sulfonic acid	2.6 ± 3.3	2.4 ± 2.6	0.99 (0.89–1.10)	0.864
2-(*N*-ethyl-PFOSA) acetate	0.2 ± 0.2	0.2 ± 0.1	1.50 (1.08–2.09)	0.020
2-(*N*-methyl-PFOSA) acetate	0.6 ± 0.8	0.6 ± 0.6	1.08 (0.96–1.22)	0.183
Perfluorodecanoic acid	0.5 ± 0.6	0.5 ± 0.9	0.98 (0.83–1.17)	0.838
Perfluorobutane sulfonic acid	0.1 ± 0.2	0.1 ± 0.1	0.98 (0.55–1.75)	0.936
Perfluoroheptanoic acid	0.4 ± 0.4	0.3 ± 0.4	0.99 (0.82–1.19)	0.887
Perfluorononanoic acid	1.3 ± 1.1	1.4 ± 1.4	1.03 (0.83–1.29)	0.747
Perfluorooctane sulfonamide	0.1 ± 0.2	0.1 ± 0.1	1.09 (0.88–1.36)	0.389
Perfluoroundecanoic acid	0.3 ± 0.5	0.3 ± 0.6	0.99 (0.82–1.19)	0.908
Perfluorododecanoic acid	0.1 ± 0.1	0.1 ± 0.1	1.72 (1.08–2.73)	0.026

Adjusted for age, sex, body mass index, ratio of family income to poverty, serum cotinine (smoking status), vitamin D level, physical activity level, urinary creatinine and subsample weighting

**Table 4:** Associations between urinary chemicals and unrested during the day in adults

	No unrested (*n* = 2639, 47.5%)	Unrested during the day (*n* = 2914, 52.5%)	OR (95% CI)	*P* value
Phenol (*n* = 1486, ng/mL)				
4-*Tert*-octylphenol	0.2 ± 0.3	0.2 ± 0.1	1.93 (1.02–3.67)	0.044
Polyfluoroalkyl compounds (*n* = 1592, μg/mL)				
Perfluorooctanoic acid	4.6 ± 3.3	4.5 ± 3.6	1.01 (0.84–1.22)	0.902
Perfluorooctane sulfonic acid	22.4 ± 19.5	20.7 ± 16.9	1.09 (0.93–1.28)	0.278
Perfluorohexane sulfonic acid	2.5 ± 2.9	2.5 ± 3.2	1.03 (0.94–1.14)	0.467
2-(*N*-ethyl-PFOSA) acetate	0.2 ± 0.1	0.2 ± 0.3	1.29 (0.82–2.00)	0.248
2-(*N*-methyl-PFOSA) acetate	0.6 ± 0.7	0.6 ± 0.7	1.24 (1.02–1.51)	0.035
Perfluorodecanoic acid	0.5 ± 0.9	0.5 ± 0.6	1.10 (0.92–1.30)	0.265
Perfluorobutane sulfonic acid	0.1 ± 0.1	0.1 ± 0.2	1.42 (1.02–1.98)	0.041
Perfluoroheptanoic acid	0.3 ± 0.3	0.4 ± 0.5	1.00 (0.77–1.31)	0.974
Perfluorononanoic acid	1.4 ± 1.3	1.4 ± 1.2	1.14 (0.95–1.38)	0.148
Perfluorooctane sulfonamide	0.1 ± 0.1	0.1 ± 0.2	1.15 (0.93–1.43)	0.176
Perfluoroundecanoic acid	0.3 ± 0.7	0.3 ± 0.4	1.08 (0.85–1.36)	0.502
Perfluorododecanoic acid	0.1 ± 0.04	0.2 ± 0.1	2.11 (0.79–5.62)	0.126

Adjusted for age, sex, body mass index, ratio of family income to poverty, serum cotinine (smoking status), vitamin D level, physical activity level, urinary creatinine and sub-sample weighting

**Table 5:** Associations between urinary chemicals and leg jerks while sleeping in adults

	No leg jerks (*n* = 4780, 86.6%)	Leg jerks while sleeping (*n* = 740, 13.4%)	OR (95% CI)	*P* value
Arsenic (*n* = 1567, μg/L)				
Total arsenic	22.1 ± 56.7	28.8 ± 106.5	1.03 (0.88–1.21)	0.714
Arsenous acid	0.9 ± 0.6	0.9 ± 0.2	0.89 (0.32–2.45)	0.802
Arsenic acid	0.8 ± 0.4	0.8 ± 0.6	0.73 (0.28–1.94)	0.508
Arsenobetaine	11.7 ± 43.4	18.5 ± 92.2	0.99 (0.87–1.13)	0.869
Arsenocholine	0.4 ± 0.3	0.5 ± 0.3	1.02 (0.60–1.70)	0.951
Dimethylarsonic acid	5.8 ± 9.5	5.2 ± 4.7	0.96 (0.80–1.14)	0.586
Monomethylarsonic acid	1.0 ± 1.2	0.9 ± 0.6	0.97 (0.60–1.58)	0.904
Trimethylarsine oxide	0.7 ± 0.2	0.7 ± 0.4	1.72 (1.09–2.72)	0.022
Polyaromatic hydrocarbons (*n* = 1412, ng/L)				
2-Hydroxyfluorene	722.2 ± 1257.0	912.4 ± 1540.5	1.21 (1.02–1.43)	0.029
3-Hydroxyfluorene	353.8 ± 880.4	461.8 ± 784.5	1.19 (1.02–1.39)	0.029
9-Hydroxyfluorene	649.8 ± 1237.2	703.3 ± 1243.4	1.21 (0.96–1.53)	0.104
1-Hydroxyphenanthrene	265.6 ± 528.0	259.6 ± 305.9	1.23 (0.97–1.55)	0.081
2-Hydroxyphenanthrene	123.4 ± 279.5	127.3 ± 174.7	1.17 (0.91–1.51)	0.207
3-Hydroxyphenanthrene	199.0 ± 486.5	223.6 ± 370.5	1.22 (0.95–1.56)	0.114
1-Hydroxypyrene	214.0 ± 627.3	279.9 ± 790.4	1.22 (1.03–1.45)	0.025
1-Hydroxynapthalene (1-naphthol)	22,657.5 ± 177,331.5	175,350.4 ± 1,968,678.0	1.05 (0.91–1.21)	0.497
2-Hydroxynapthalene (2-naphthol)	9312.6 ± 25,015.5	10,078.5 ± 14,846.3	1.02 (0.80–1.30)	0.849
4-Hydroxyphenanthrene	54.9 ± 99.4	50.6 ± 52.5	1.06 (0.85–1.33)	0.575
Polyfluoroalkyl compounds (*n* = 1584, μg/mL)				
Perfluorooctanoic acid	4.5 ± 3.3	4.7 ± 4.1	1.05 (0.89–1.24)	0.523
Perfluorooctane sulfonic acid	21.4 ± 18.0	22.2 ± 19.0	1.05 (0.90–1.24)	0.488
Perfluorohexane sulfonic acid	2.5 ± 3.0	2.8 ± 3.1	1.06 (0.87–1.31)	0.528
2-(*N*-ethyl-PFOSA) acetate	0.2 ± 0.2	0.2 ± 0.1	0.94 (0.47–1.91)	0.867
2-(*N*-methyl-PFOSA) acetate	0.6 ± 0.7	0.6 ± 0.5	1.27 (1.12–1.44)	0.001
Perfluorodecanoic acid	0.5 ± 0.7	0.6 ± 0.9	1.03 (0.84–1.27)	0.744
Perfluorobutane sulfonic acid	0.1 ± 0.2	0.1 ± 0.1	1.65 (1.03–2.66)	0.040
Perfluoroheptanoic acid	0.3 ± 0.4	0.4 ± 0.7	1.26 (1.02–1.56)	0.035
Perfluorononanoic acid	1.4 ± 1.3	1.3 ± 1.2	1.05 (0.82–1.33)	0.694
Perfluorooctane sulfonamide	0.1 ± 0.2	0.1 ± 0.1	0.89 (0.66–1.21)	0.448
Perfluoroundecanoic acid	0.3 ± 0.5	0.3 ± 0.6	0.97 (0.77–1.21)	0.746
Perfluorododecanoic acid	0.1 ± 0.1	0.1 ± 0.1	1.39 (0.55–3.52)	0.466

Adjusted for age, sex, body mass index, ratio of family income to poverty, serum cotinine (smoking status), vitamin D level, physical activity level, urinary creatinine and sub-sample weighting

**Table 6:** Associations between urinary chemicals and leg cramps while sleeping in adults

	No leg cramps (*n* = 4496, 80.9%)	Leg cramps while sleeping (*n* = 1059, 19.1%)	OR (95% CI)	*P* value
Pesticides (*n* = 1489, μg/L)				
2,5-dichlorophenol	228.2 ± 1242.6	175.0 ± 772.2	1.12 (1.02–1.23)	0.018
*O*-phenyl phenol	0.2 ± 0.6	0.3 ± 2.2	0.98 (0.72–1.35)	0.904
2,4-Dichlorophenol	7.4 ± 45.9	5.5 ± 24.0	1.17 (1.03–1.32)	0.017
2,4,5-Trichlorophenol	0.1 ± 0.3	0.1 ± 0.1	1.08 (0.86–1.37)	0.466
2,4,6-Trichlorophenol	0.7 ± 2.7	0.7 ± 2.4	0.91 (0.67–1.23)	0.511
Heavy metals (*n* = 1576, μg/L)				
Barium	2.3 ± 3.9	2.2 ± 3.2	0.93 (0.81–1.06)	0.257
Cadmium	0.4 ± 0.4	0.5 ± 0.5	1.05 (0.91–1.21)	0.463
Cobalt	0.5 ± 1.2	0.8 ± 2.3	1.07 (0.87–1.30)	0.506
Cesium	2.3 ± 3.9	2.2 ± 3.2	0.92 (0.78–1.08)	0.278
Molybdenum	5.7 ± 4.0	5.4 ± 4.5	0.98 (0.79–1.20)	0.813
Lead	61.1 ± 53.1	57.7 ± 51.1	1.02 (0.80–1.29)	0.895
Antimony	1.0 ± 2.3	0.9 ± 0.8	1.22 (1.05–1.43)	0.015
Thallium	0.2 ± 1.3	0.1 ± 0.2	0.87 (0.72–1.05)	0.136
Tungsten	0.2 ± 0.1	0.2 ± 0.1	1.07 (0.91–1.26)	0.392
Uranium	0.1 ± 0.3	0.2 ± 0.4	1.27 (1.06–1.51)	0.011
Polyaromatic hydrocarbons (*n* = 1425, ng/L)				
2-Hydroxyfluorene	738.5 ± 1275.0	764.3 ± 1373.7	1.19 (1.00–1.43)	0.054
3-Hydroxyfluorene	371.7 ± 904.2	340.3 ± 676.4	1.14 (0.96–1.36)	0.121
9-Hydroxyfluorene	644.6 ± 1212.2	692.2 ± 1320.2	1.28 (1.12–1.45)	0.001
1-Hydroxyphenanthrene	259.3 ± 520.7	284.1 ± 419.3	1.21 (0.98–1.50)	0.074
2-Hydroxyphenanthrene	121.5 ± 281.9	131.4 ± 191.6	1.24 (1.00–1.53)	0.046
3-Hydroxyphenanthrene	203.7 ± 499.2	189.1 ± 322.1	1.18 (0.97–1.43)	0.086
1-Hydroxypyrene	215.3 ± 639.9	245.8 ± 676.3	1.28 (1.10–1.50)	0.004
1-Hydroxynapthalene (1-naphthol)	23,290.6 ± 182,622.3	124,604.9 ± 1,616,734.0	1.05 (0.94–1.16)	0.368
2-Hydroxynapthalene (2-naphthol)	8859.9 ± 20,058.9	11,969.1 ± 36,521.9	1.14 (0.97–1.33)	0.104
4-Hydroxyphenanthrene	53.8 ± 97.5	55.6 ± 80.6	1.15 (0.92–1.43)	0.214
Phthalate (*n* = 1488, μg/mL)				
Mono(carboxyoctyl)	17.8 ± 106.8	11.5 ± 20.3	1.10 (0.95–1.28)	0.174
Mono(carboxynonyl)	6.2 ± 25.8	5.3 ± 12.5	1.15 (1.02–1.31)	0.025
Mono-2-ethyl-5-carboxypentyl	103.8 ± 313.6	105.2 ± 291.9	1.06 (0.90–1.24)	0.489
Mono-*n*-butyl	41.7 ± 272.7	46.1 ± 162.8	1.13 (0.97–1.31)	0.100
Mono(3-carboxypropyl)	4.3 ± 14.7	3.9 ± 6.0	1.20 (1.02–1.42)	0.032
Monoethyl	480.7 ± 1212.1	404.0 ± 912.0	1.06 (0.98–1.14)	0.141
Mono(2-ethyl-5-hydroxyhexyl)	81.6 ± 280.8	75.6 ± 219.3	1.06 (0.92–1.21)	0.412
Mono(2-ethyl)-hexyl	12.4 ± 62.8	9.2 ± 26.6	1.06 (0.93–1.21)	0.360
Monoisobutyl	21.4 ± 397.3	11.7 ± 23.5	1.12 (1.00–1.26)	0.057
Mono-*n*-methyl	4.3 ± 27.5	4.9 ± 23.5	1.03 (0.87–1.21)	0.735
Monoisononyl	2.1 ± 8.2	1.2 ± 1.3	1.07 (0.81–1.43)	0.605
Mono(2-ethyl-5-oxohexyl)	50.5 ± 177.8	46.9 ± 136.1	1.04 (0.90–1.19)	0.587
Monobenzyl	29.3 ± 451.3	20.3 ± 45.1	1.06 (0.93–1.19)	0.357
Monocyclohexyl	0.5 ± 0.5	0.5 ± 1.7	1.38 (0.67–2.85)	0.353

Adjusted for age, sex, body mass index, ratio of family income to poverty, serum cotinine (smoking status), vitamin D level, physical activity level, urinary creatinine and subsample weighting

## Discussion

### Arsenic and sleep

Low level of arsenic exposure was previously observed to be associated with sleep disturbance in copper smelter workers (*n* = 680; Lilis et al. 1985). Long-term poisoning was also found to affect sleep disorder in children (Ishi and Tamaoka 2015). In the present study, higher levels of urinary arsenic were found to be associated with wake-up at night and leg jerk while sleeping. However, to date no experimental research was available to confirm the biological mechanism yet.

### Pesticides and sleep

Pesticide exposure, particularly 2,4,5-trichlorophenol, was recently found to influence idiopathic REM sleep behavior disorder in older adults (*n* = 694; Postuma et al. 2012; Neuberger et al. 1998). In the limited experimental research, exposure to pesticides seemed to reduce sleep time in male mice (21–24 g; Chaturvedi 1993). In the present study using national representative human sample, 2,5-dichlorophenol and 2,4-dichlorophenol showed borderline associations with leg cramps in sleeping. This would need future longitudinal or experimental research to confirm or refute the findings in order to rule out the statistical significance by chance.

### Heavy metals and sleep

Uranium might directly affect the brain. Previously, it was observed that chronic uranium exposure (40 mg l(−1) in drinking water, for 90 days) increased in rapid eye movement sleep in rats (Lestaevel et al. 2005). In the present study, the consistent finding was shown in higher levels of urinary uranium and urinary antimony and leg cramps in sleeping.

### Phthalates and sleep

The intravenous or intraperitoneal administration of bis(2-ethylhexyl) phthalate could prolong hexobarbital sleep time in mice and rats by enlarging the lipophilic pool (Swinyard et al. 1976). In the present study, monocyclohexyl showed an association with wake-up at night, and mono(carboxynonyl) and mono(3-carboxypropyl) showed associations with leg cramps in sleeping. These would also need future longitudinal or experimental research to confirm or refute the findings in order to rule out the statistical significance by chance.

### Polyaromatic hydrocarbons and sleep

There was no direct research evidence on the relationship of polyaromatic hydrocarbons and sleep health. In the present study, it is the first time to show that higher urinary polyaromatic hydrocarbons, such as 2-hydroxyfluorene, 9-hydroxyfluorene, 2-hydroxyphenanthrene, and 1-hydroxypyrene, were significantly associated with leg cramps in sleeping. Similarly, urinary 2-hydroxyfluorene, 3-hydroxyfluorene, 1-hydroxypyrene were significantly associated with leg jerks in sleeping. Levels of other known urinary polyaromatic hydrocarbons were also higher in people with sleep disturbances, but the statistical significance was not reached.

### Polyfluoroalkyl compounds and sleep

There were no indications that there could be any association between polyfluoroalkyl compounds and sleep health in the past. Polyfluoroalkyl compounds in human health is an under-studied area, mostly with a focus on fetal growth, neuro-behavioral problems in infants or children, or cognitive function in older adults (Bach et al. 2015; Donauer et al. 2015; Power et al. 2013; Maisonet et al. 2012; Hoffman et al. 2010). In the present study using national representative human sample, the new observations have consistently shown higher levels of urinary 2-(*N*-ethyl-PFOSA) acetate and perfluorododecanoic acid in people with wake-up at night, higher levels of urinary 2-(*N*-methyl-PFOSA) acetate, perfluorobutane sulfonic acid in people feeling unrested during the day, and higher levels of 2-(*N*-methyl-PFOSA) acetate, perfluorobutane sulfonic acid and perfluoroheptanoic acid in people with leg jerks in sleeping. However, to date there has been no experimental research to confirm yet.

### Strengths and limitations

The present study has a few strengths. Firstly, this study was conducted in a large and nationally representative human sample with mixed ethnicities and socioeconomic status. Secondly, this was the first time to examine the risk associations between different sets of environmental chemical concentrations and common sleep troubles. However, there are still some limitations that cannot be ignored. First, there could be still other emerging chemicals from the living environments that we might not yet know and would need future research to further identify and examine. Second, objective measurements on sleep patterns were not available. Third, the causality cannot be established in the present study due to the cross-sectional study design in nature. Therefore, future studies with longitudinal or experimental study designs to confirm or refute the current findings and, if at all, to understand the persisting risk effects along the life course from those mentioned above environmental chemicals would be warranted.

### Directions for future research, practice, and policy

Urinary arsenic, pesticides, heavy metals, phthalates, polyaromatic hydrocarbons and polyfluoroalkyl compounds were associated with common sleep troubles in adults For future research, studies understanding the biological mechanism and monitoring the risk effects along the life course with a longitudinal or experimental approach would be needed. For practice and policy, elimination of these environmental chemicals in order to improve sleep health might be considered.
